# Coexistence of Lichen Planus and Squamous Cell Carcinoma on Sun-Exposed Skin: A Case Report

**DOI:** 10.7759/cureus.86194

**Published:** 2025-06-17

**Authors:** Adelina Velikova, Anastasia I Bekyarova, Andreya Kirilova, Kristina Naydenova, George S Stoyanov, Hristo Popov

**Affiliations:** 1 General and Clinical Pathology, Forensic Medicine and Deontology, Medical University - Varna, Varna, BGR; 2 Pathology, Multiprofile Hospital for Active Treatment, Shumen, BGR

**Keywords:** actinic keratosis, cutaneous squamous cell carcinoma, lichen planus, skin, solar elastosis, squamous cell carcinoma, sun exposure

## Abstract

Lichen planus (LP) is a chronic idiopathic skin disorder that has a predilection for sun-exposed areas. Although rare, there is a possibility that chronic LP lesions could progress to squamous cell carcinoma (SCC), a non-melanocytic malignant skin neoplasm. Cumulative exposure to ultraviolet radiation, particularly in individuals with lighter skin tones, is one of the leading risk factors for cutaneous SCC (cSCC). In this report, we present a case of a 79-year-old male patient who was diagnosed with a moderately differentiated cSCC with facial localization. This condition developed on the background of preexisting actinic keratosis, and was accompanied by a LP.

## Introduction

Lichen planus (LP) is a chronic idiopathic skin condition affecting the skin, mucous membranes, hair follicles, and nail plates. Classic cutaneous eruptions manifest as the "six Ps": purple, pruritic, planar, polygonal papules, and plaques. They are located on the wrists, lower back, and legs with a predilection for areas of the body that are frequently exposed to sunlight [1,2]. The most common clinical subtypes of cutaneous LP include hypertrophic LP (HLP), ulcerative LP, pigmented LP, actinic LP, and bullous LP. Mucosal LP is characterized by the presence of erosions, predominantly within the oral cavity, which are associated with pain and burning sensations [1,2]. LP is most commonly observed in middle-aged adults, with an estimated incidence in the United States of approximately 150 cases per 100,000 population [3]. The etiology of LP remains unclear, although there are predisposing factors, including hepatitis C virus (HCV) and antihypertensive medications such as angiotensin-converting enzyme inhibitors (ACEIs) and thiazide diuretics, that may be associated with the condition. According to the European guidelines on the management of LP, the treatment of choice is topical glucocorticoids [4]. Persistent lesions are regarded as possible precursors to squamous cell carcinoma (SCC) [2].

Cutaneous squamous cell carcinoma (cSCC) is a malignant skin neoplasm of epidermal keratinocytes that occurs in the same anatomical locations and age group as LP [5]. In contrast to LP, cSCC has well-established etiopathological factors, including cumulative exposure to ultraviolet (UV) radiation, particularly in individuals with lighter skin tones, human papillomavirus (HPV) infection, radiation from X-rays, certain genetic syndromes, middle age, and chronic non-healing skin lesions such as burn wounds and actinic keratosis (AK) [6,7].

## Case presentation

Herein, we present a case of a 79-year-old male patient with a medical history of long-standing hypertension and arrhythmia, adequately managed with olmesartan, amiodarone, verapamil, and edoxaban. The patient was unable to provide precise information regarding both the duration and the dosage of his treatment with the aforementioned medications. He had never had cancer or a family history of cancer. 

**Figure 1:**
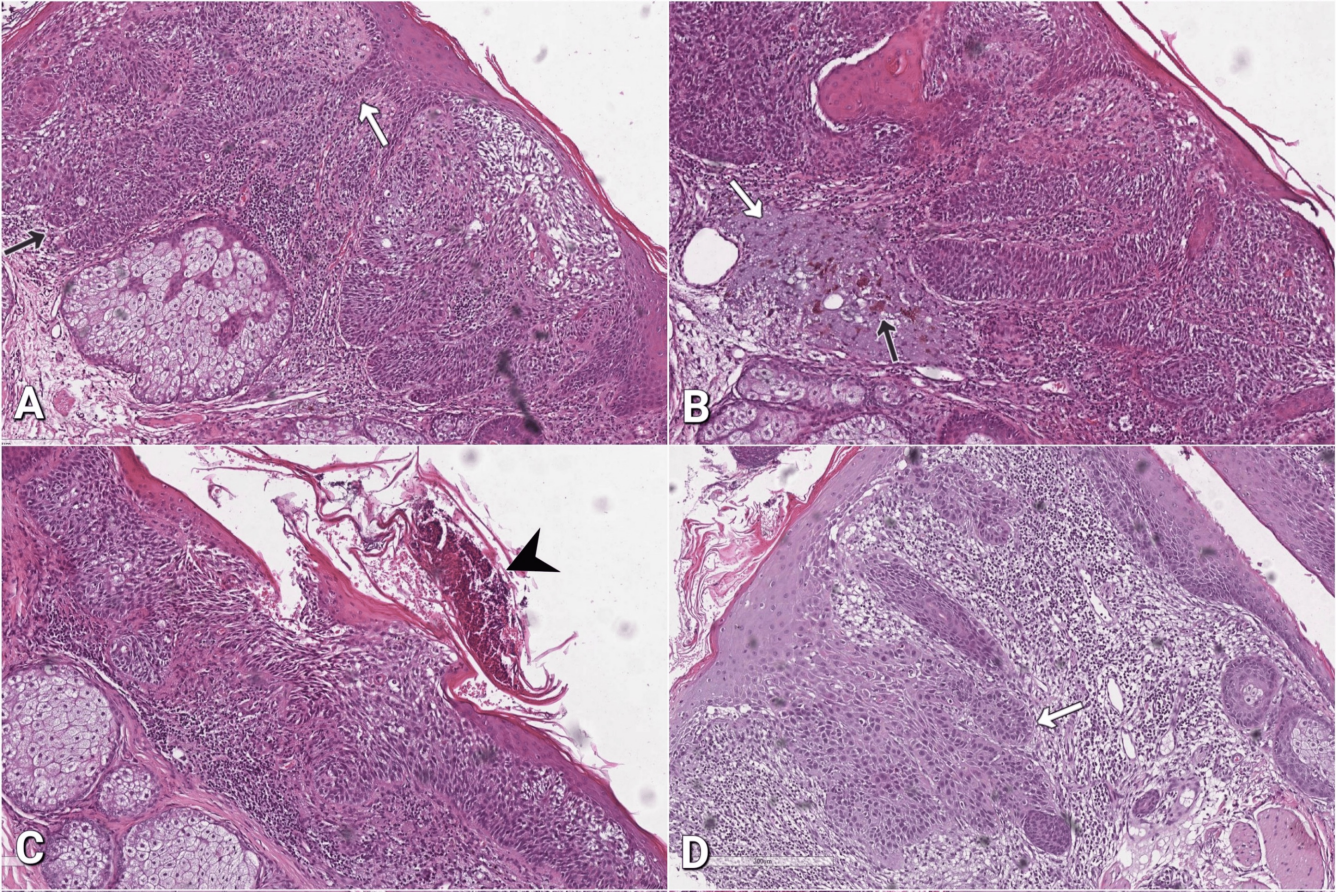
Squamous cell carcinoma and adjacent changes under hematoxylin and eosin (H&E) stain (A) Cords of atypical cells extending from the epidermis (white arrow) and forming nests in the papillary derma (black arrow), 100x; (B) Thickened layer of blue-gray elastic fibers with amorphous appearance, representing solar elastosis (white arrow) and pigment incontinence (black arrow) within the dermis, 100x; (C) Ulcerative focus with debris (arrowhead), 100x; (D) Pseudoepitheliomatous hyperplasia (white arrow), 100x.

The patient presented with two facial skin lesions with ulcerative changes that had appeared consecutively in close vicinity. In the last few days before admission to the hospital, he experienced local pain and noticed marked redness surrounding the lesions. He could not specify the moment of the initial appearance of the lesions, nor their subsequent evolution. Furthermore, no systemic monitoring by a dermatologist had been conducted. A consultation with a surgeon found ulcerative changes in the problematic cutaneous region, and the patient was subsequently referred for surgical treatment. A complete excision of the lesions was performed under local anesthesia, and the specimen was examined histopathologically. Grossly, the skin specimen measuring 13×6 mm contained two lesions with an exophytic growth pattern and a granular, lobulated whitish appearance. Histologic sections revealed epidermis composed of atypical cells with large, prominent hyperchromatic nucleoli, forming discrete nests with focal keratinization (Figure 1A).

The tumor cells invaded the papillary dermis, where solar elastosis and pigment incontinence were also present (Figure 1B). The neoplasm was identified as moderately-differentiated SCC.

Superficially, an area showed ulcerative changes with hemorrhagic and detritic components (Figure 1C). The tumor tissue was accompanied by the presence of pseudoepitheliomatous hyperplasia, which was characterized by the thickening of the overlying epithelium without significant nuclear atypia (Figure 1D).

**Figure 2:**
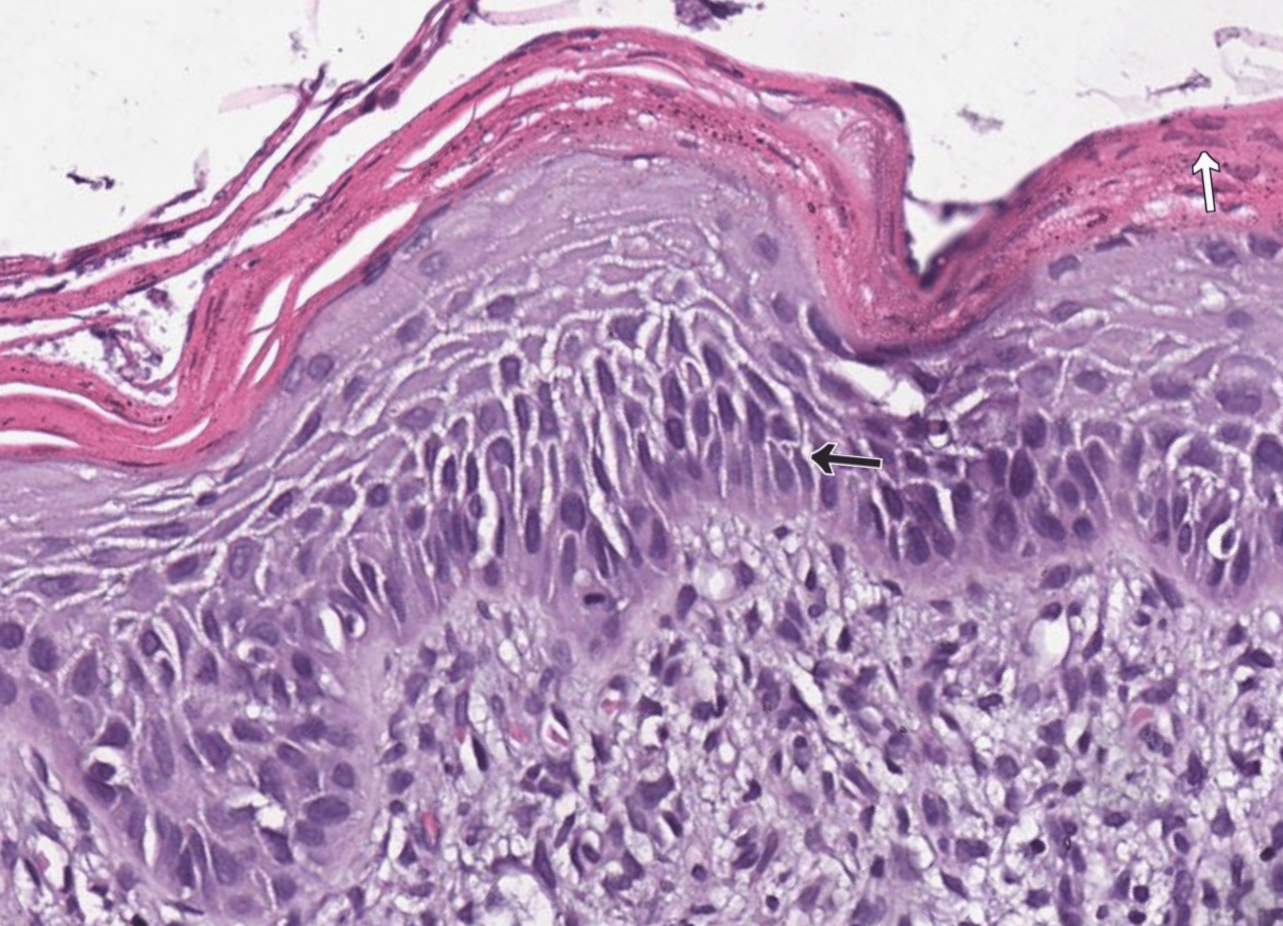
Actinic keratosis under hematoxylin and eosin (H&E) stain Atypical basal keratinocytes with loss of polarization (black arrow), and mild crowding, reaching the mid epidermal layers, focal parakeratosis (white arrow), and a downward bud in the left lower quadrant of the image, 200x.

Adjacent to the tumor tissue, AK (Figure 2), demonstrating basal atypia with loss of polarization, mild crowding, nuclear pleomorphism and hyperchromasia, focal parakeratosis, downward buds, and loss of the granular layer was noted.

Based on the histopathological findings, we suggest that the SCC developed on the background of preexisting AK.

**Figure 3:**
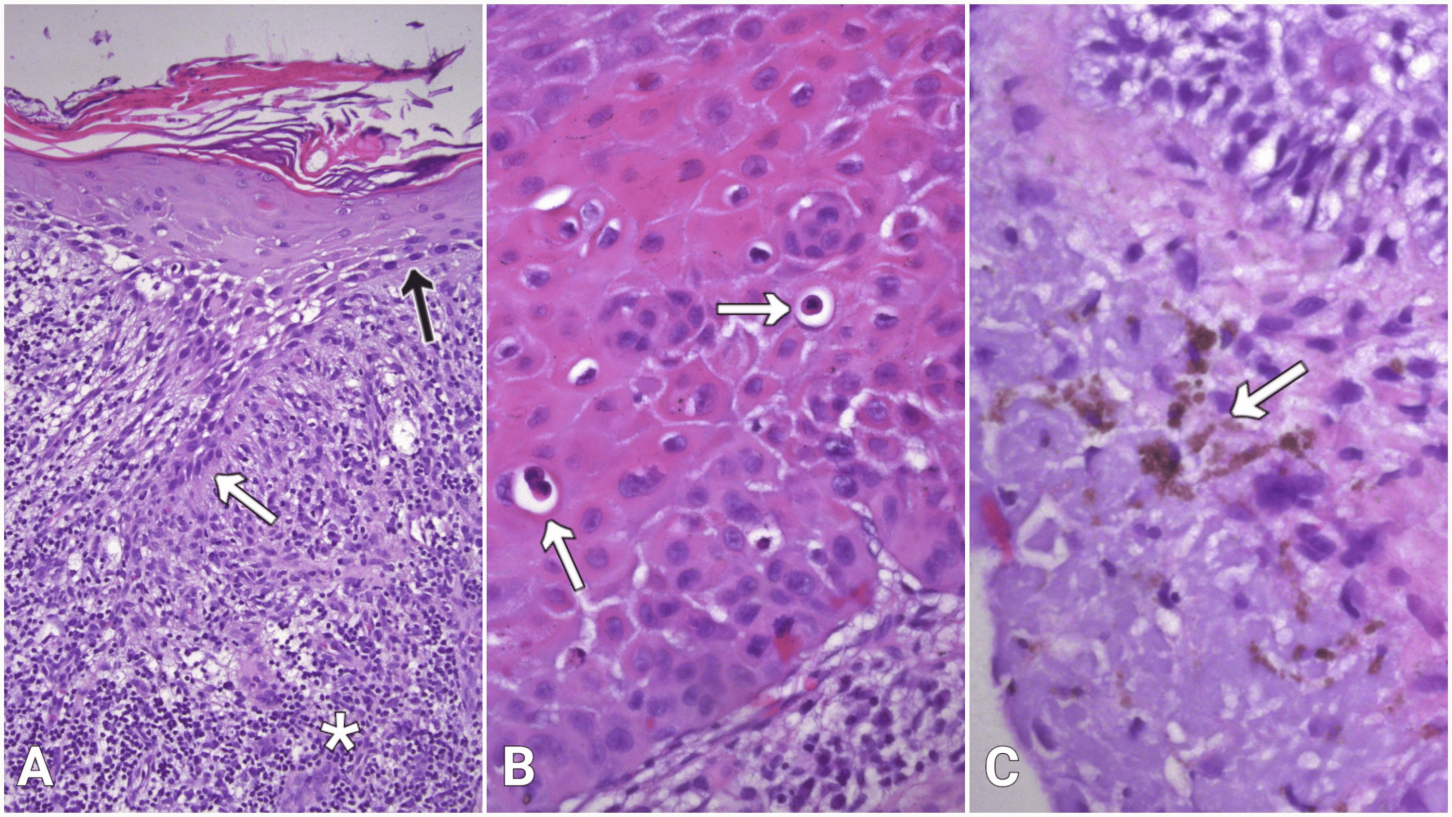
Histological features of lichen planus under hematoxylin and eosin (H&E) stain (A) Sawtoothed rete ridges (white arrow), surrounded by dermal lymphohistiocytic infiltrate (asterisk); flattened epidermal basal cells (black arrow) and basal cells with hydropic degeneration, 100х; (B) Multiple Civatte bodies (arrows) within the epidermis, 400x; (C) Pigment incontinence in the upper dermis (arrow), 400х.

In close vicinity to the AK, the epidermis displayed zones of hypergranulosis and Civatte bodies, accompanied by mildly sawtoothed rete ridges, a band-like dermal lymphohistiocytic infiltrate, and pigment incontinence (Figure 3).

The mentioned features are considered diagnostic for LP. The resection margins were negative for SCC and LP. 

The patient did not report any additional risk factors that might have contributed to the development of SCC in a LP lesion.

## Discussion

Mucosal LP, more specifically oral LP, has proven to be an oncogenic lesion [8,9]. In contrast, the evidence for cutaneous LP as a premalignant lesion is inconclusive. The development of SCC in an LP lesion is rare, with a prevalence of 0.4% among patients, with an average time of 12 years for LP to evolve into SCC [10,11]. Furthermore, SCC is more common in patients diagnosed with HLP than in those with simple LP. The pathogenesis of SCC in HLP is hypothesized to be the result of chronic antigen stimulation and accelerated cell turnover [10]. A comprehensive analysis of 38 cases revealed that the majority of cSCCs arising in HLP were well-differentiated variants, encompassing verrucous carcinoma and keratoacanthoma subtypes [12]. It is important to note that there is a lack of consensus within the scientific community regarding the classification of keratoacanthoma as either a well-differentiated SCC or a precancerous lesion. The development of SCC in the presence of LP may be influenced by a number of factors, including UV radiation, chronic inflammation, and tar application [13]. The patient in this case did not report any of the aforementioned factors, and the duration of the LP lesion was unknown. In addition, the aforementioned antiarrhythmic and antihypertensive medications, which the patient was taking, have been observed to induce lichenoid-like reactions. From both a clinical and a histological perspective, these reactions may be indistinguishable from classic LP [2]. Given its photosensitivity, actinic LP should be considered as a possible differential diagnosis in our case, as it occurs primarily on sun-exposed areas. While the precise pathogenesis of actinic LP is not yet fully comprehended, UV radiation has been identified as a significant inciting factor. It is proposed that UV radiation plays a critical role in the expression of altered self-antigens on basal keratinocytes, thereby enabling the recruitment of cytotoxic T-cells and the subsequent occurrence of characteristic histopathological changes.

In certain cases, LP may present with clinical features such as scaly, hyperkeratotic plaques that resemble SCC. However, histopathological examination usually reveals HLP with pseudoepitheliomatous hyperplasia, but no evidence of a malignancy [11,14]. Reported LP lesions with malignant transformation into SCC are predominantly located on the pretibial region of the lower limb [10-12,14]. This area is relatively often exposed to sunlight and easily accessible for scratching, considering the pruritic nature of the dermatosis. The case presented here concerns a coexistence of LP and SCC, located on the face, which to the best of our knowledge, is the first documented case of its nature. There is, however, one previously reported case of basal cell carcinoma in the paranasal region developing from a long-standing cutaneous LP [15]. A differential diagnosis is required between cutaneous LP and benign lichenoid keratosis (BLK). The distinguishing feature between the two conditions is that BLK most commonly manifests as a solitary lesion, whereas LP typically presents as multiple lesions [16].

Regarding our case, the facial localization is closely related to chronic UV light exposure, which is morphologically demonstrated by the presence of solar elastosis in the dermis and initially carries a higher risk of malignancies. Keeping this in mind, it is imperative to consider whether LP served as a precursor lesion in the development of SCC or was it simply a coincidental coexistence in this particular case. 

In light of the observed pseudoepitheliomatous hyperplasia, the hypothesis that this case may represent a coexistence of HLP and pseudoepitheliomatous hyperplasia is a plausible one. However, SCC is more prevalent in the histologic sections and the LP does not fulfil the criteria for HLP.

## Conclusions

We highlight the potential association between LP and SCC on chronically sun-exposed skin, a rare condition whose etiopathogenesis remains to be elucidated. The question of whether LP and SCC have a cause-and-effect relationship or if they are merely colocalized remains speculative at this point in time. Further research is required to determine the validity of these assumptions, and both possibilities remain open until more cases are reported. Clarification of this issue could have an impact on future monitoring and treatment strategies for LP in high-risk areas. 
